# Monitoring canid scent marking in space and time using a biologging and machine learning approach

**DOI:** 10.1038/s41598-019-57198-w

**Published:** 2020-01-17

**Authors:** Owen R. Bidder, Agustina di Virgilio, Jennifer S. Hunter, Alex McInturff, Kaitlyn M. Gaynor, Alison M. Smith, Janelle Dorcy, Frank Rosell

**Affiliations:** 1 Department for Environmental Science, Policy and Management, University of California, Berkeley, United States of America; 2 Grupo de Ecología Cuantitativa, INIBIOMA (UnComa-CONICET), Bariloche, Río Negro Argentina; 3 Hopland Research and Extension Center, University of California, Division of Agriculture and Natural Resources, California, United States of America; 4 Faculty of Technology, Natural Sciences, and Maritime Sciences, Department of Natural Sciences and Environmental Health, University of South-Eastern Norway, Bø i Telemark, Norway

**Keywords:** Behavioural ecology, Ecophysiology

## Abstract

For canid species, scent marking plays a critical role in territoriality, social dynamics, and reproduction. However, due in part to human dependence on vision as our primary sensory modality, research on olfactory communication is hampered by a lack of tractable methods. In this study, we leverage a powerful biologging approach, using accelerometers in concert with GPS loggers to monitor and describe scent-marking events in time and space. We performed a validation experiment with domestic dogs, monitoring them by video concurrently with the novel biologging approach. We attached an accelerometer to the pelvis of 31 dogs (19 males and 12 females), detecting raised-leg and squat posture urinations by monitoring the change in device orientation. We then deployed this technique to describe the scent marking activity of 3 guardian dogs as they defend livestock from coyote depredation in California, providing an example use-case for the technique. During validation, the algorithm correctly classified 92% of accelerometer readings. High performance was partly due to the conspicuous signatures of archetypal raised-leg postures in the accelerometer data. Accuracy did not vary with the weight, age, and sex of the dogs, resulting in a method that is broadly applicable across canid species’ morphologies. We also used models trained on each individual to detect scent marking of others to emulate the use of captive surrogates for model training. We observed no relationship between the similarity in body weight between the dog pairs and the overall accuracy of predictions, although models performed best when trained and tested on the same individual. We discuss how existing methods in the field of movement ecology can be extended to use this exciting new data type. This paper represents an important first step in opening new avenues of research by leveraging the power of modern-technologies and machine-learning to this field.

## Introduction

Scent marking is a critically important form of olfactory communication, shaping affiliative and agonistic interactions within and across species. The use of scent marks provides animals a means to defend resources^1,2^, advertise availability to mates^3–5^, and communicate with conspecifics^6,7^. It also serves to minimise the risk of injuries from agonistic encounters with potential competitors^2^. Prey species are also known to ‘eavesdrop’ on these communications to avoid contact with their predators^8^. Thus, scent marking is a critical communication system, the nuances of which dictate where animals occur on the landscape, with implications for individual fitness and, ultimately, population dynamics. Despite its importance, research on this and other forms of olfactory communication in the wild is hampered by a lack of tractable methods. This is due in part to human dependence on vision as our primary sensory modality. If new methods could be developed that address the deficiencies in our olfactory senses using modern technology, this could open up numerous avenues for future research on this significant yet under-served ecological strategy.

## Scent Marking in Canids

Scent marking is particularly important for canid species, which rely heavily on olfactory cues for sociality and territorial defence^9^. For territory holders, conspicuous advertisement through scent marking is a convenient, low-cost form of defence compared to frequent, risky physical escalation^10^. This active defence distinguishes a territory from a home-range^11^. Animals gain some benefits from visiting areas outside their territory, e.g. information on the distribution and reproductive receptiveness of neighbours^12^, but they will obtain the most direct fitness benefits from resources within their territories, for which they will have almost exclusive use^2,13^. However, extra-territorial movements (i.e. ‘forays’^14^) can be indicative of other important ecological processes. In wolves for example, extra-territorial forays may be performed by entire packs in response to scarcity of resources, or by individuals as failed dispersal attempts^15,16^. As such, information about how much of an animal’s home range constitutes its territory, and their time spent in and outside that territory, is germane to our understanding of these animals’ ecology. These topics can only be investigated by establishing the spatial distribution of scent marks in the landscape.

Temporal variation of scent marks efficacy is also a pertinent issue that is incorporated in theoretical treatise of territoriality and spatial resource partitioning^17–19^. However, the signal decay due to precipitation, ultraviolet radiation and bacterial decomposition has rarely been investigated empirically^20,21^. For example, free-ranging dogs have been observed to scent mark more during the wet season^22^, thus increasing the cost of territory defence at these times. It is possible that changes in rainfall or ultraviolet radiation, due to climate change for instance^23^, may have an impact on the economics of territory defence for scent marking species more generally^10^. Thus, in addition to information on their spatial distribution, records of when animals lay scent marks, which conspecifics come in to contact with these signals and for how long they are detected, are all pertinent and merit study.

## Limited Methods

Gaining information about the when and where of scent marking of wild animals is a non-trivial methodological issue. Camera traps provide a means to study free-living animals and have provided valuable insights for wild canids^24,25^. However, these animals are prompted to scent-mark by a stimulus placed by the researcher, and so do not offer insight into the typical spatial distribution of scent marks or the nuances of their use in intra-species communication. Following individuals for direct observation has provided some insights on spatial distribution and the environmental context for scent marking^26^, but this method may suffer from observer bias^27,28^, limit observations to small areas^29^, and is labour intensive. To study the spatial distribution of scent marking, researchers have been limited to tracking field signs in snow^30–33^. This method may not provide information on animal identity that is important for social species, as their scent marking activity and strategy may differ according to factors such their place in the social hierarchy^34^. Tracking in the snow is also restrictive because it cannot be used in warm climates or at times of year in which snow is absent.

In summary, our ability to study the dynamics of scent marking has been greatly limited due to the challenges of measuring olfactory marks in the field. Studies of animal communication have been biased towards more easily detectable visual and auditory signals, and there are few existing tools to study olfactory communication. These limitations are particularly acute for canids as they can be cryptic, travel large distances over difficult terrain, operate nocturnally, and rely heavily on olfactory communication. Currently, researchers are unable to obtain adequate samples, precluding studies on species that are difficult to observe visually. Novel functional field methods for studying olfactory communication can therefore create new opportunities for inference and understanding in ecology.

## A Novel Approach Using Biologging Techniques

Numerous studies in behavioural ecology have benefitted from advancements in the field of biologging^35^. Biologging techniques make use of miniaturized animal-borne technologies, such as heart rate monitors^36^, pressure sensors^37^ and temperature loggers^38,39^ to chronicle free-ranging animals’ physiology, behaviour and environment remotely. Accelerometers are a particularly valuable tool for monitoring animal behaviour, and are well-suited to study the distinctive movement patterns associated with canid scent marking^40^. Accelerometers record animal movement at high sample rates (typically over 25 Hz) via tri-axial (i.e. 3-dimensional) inertial sensors (see^41^ and papers therein). The large datasets these high resolution sensors produce can be analysed using machine learning techniques to quickly and automatically find behaviours of interest^41–44^. One such machine learning technique, the k-Nearest Neighbour (KNN) algorithm, is a conceptually simple and effective method of classifying behaviours in accelerometer data^45^. Many male canids are renowned for the archetypal ‘leg up’ posture that they adopt when scent marking with urine^46^, and such a static posture should be relatively conspicuous in signals produced by an accelerometer for detection by KNN. Females often adopt a ‘squat’ posture when performing urine scent marks, but whether the signal produced in this posture could be discerned from a similar posture adopted when defecating has yet to be established. Scent marking has not yet been studied by concurrent use of GPS and accelerometers (see^47^ for an example on livestock elimination events), but doing so may allow researchers to assign both time and location to scent mark observations, addressing the current methodological limitations identified above.

At this stage, it is vital to validate candidate methods that can discern when scent marking events occur. In this study, we investigate whether accelerometers and KNN analysis perform with requisite accuracy, and we integrate these technologies with GPS telemetry to describe the spatial distribution of scent marking behaviour with a biologging approach for the first time. We aim to address the deficiencies of existing methods, and introduce a novel method without the need for labour intensive human observation and that is not contingent on environmental conditions for its use. We validate this method using domestic dogs as a tractable model, recording their scent marks whilst under both accelerometer and video observation. To ensure that the method is broadly applicable across canid species, we use a variety of dog breeds that approximate the range of body sizes observed in wild canid species^48^. To further illustrate the utility of the method, and to provide the means to link scent marking records to animal location data, we apply the technique in a field trial using free-ranging guardian dogs. Using this case study as an example, we relate how novel information on scent marks in time and space may be used to address pressing questions in animal ecology, namely; how much of their home-range constitute territory for which they have exclusive use, how animals may perform extra-territorial movements to access mates and resources, and how scent mark efficacy decays over time.

## Methods

### Validation experiment

We performed a comprehensive validation study of the proposed scent marking biologging method at the University of South-Eastern Norway, in Bø, Norway (59°24N, 9°03E). The validation study took place in March 2015, around a mixed path of concrete walkway and short, firm grass turf. We obtained a sample of 62 domestic dogs, *Canis lupus familiaris* (32 female, 30 males), identified through volunteers that were members of a local interest group. All experiments were conducted in accordance with the guidelines and regulations at the University of South-Eastern Norway (other ethical approvals were not required), and all owners provided written informed consent and remained with their animals at all times. To ensure that the method was broadly applicable across species, we used breeds that varied in body size (2.6 kg to 40 kg) to emulate the range of body sizes and morphology observed in wild canids^48^. We also recorded animal weight, age and sex and tested for a relationship between these characteristics and classification accuracy. Dogs were a variety of breeds (both purebred and mix) and a mean age of 5.5 years (±3.78 years), mean weight of 17.8 kg (±10.6 kg), although details (i.e. breed and age) for 10 of the dogs were not known to the owners. Only 1 of the 30 male dogs was castrated. We equipped the dogs with a X6-mini accelerometer from Gulf Coast Data Concepts (Waveland, MS, USA), set to record at 50 Hz on 1 Gb of memory. The device weighed 18 g, well under the 5% body weight ethical standard^49,50^. Devices were positioned on the lower dorsal portion using a flexible silastic harness (Thomson Bros. Ltd., Newcastle upon Tyne, UK) designed for this purpose. Dogs were taken along a path, where they were allowed to roam freely, and were monitored via a GoPro™ camera until they had scent marked at least 5 times, with at least one instance of each left and right leg raised scent marks for male dogs, and at least one instance of squat scent mark and squat defaecation for females. Sessions ranged from 13 to 50 minutes, with sessions terminated if dogs did not perform the requisite number of scent marks within 50 minutes. Females performed predominantly squat urinations, but those that also used raised-leg urinations had those postures included in their analysis. We included defaecations as a class in the analysis for males if the animal conducted this behaviour whilst under observation. All other behaviours (e.g. walking, running, sitting) were grouped in to a single behavioural class, termed ‘other’. These other behaviours can be discerned from each other by KNN^45^, but were not the focus of this work. KNN predictions were compared to the actual behaviour as verified by the video recording, and accuracy, precision and recall, f1 score and area under the receiver operator characteristic curve (AUC) calculated (see below).

### Application experiment

The second part of our work, in which we applied the method to free-ranging canids, was conducted at the Hopland Research and Extension Center (HREC; 39°00N 123°05W), in Mendocino County, California, USA. All experiments were conducted in accordance with the guidelines and regulations of HREC and after approval from the HREC Animal Care and Use Committee. The vegetation is characteristic of the Coast Range, composed mainly of annual grassland, chaparral, oak woodland and mixed evergreen-deciduous forest. HREC is a University of California field research facility for agriculture and natural resources, and at present maintains a research sheep flock of between 500 and 600 breeding ewes. Losses of sheep by coyote (*C. latrans*) are an issue^51^. To reduce the need for lethal control, the HREC uses 5 guardian dogs (e.g.^52^) to discourage predation of sheep by coyotes. These guardian dogs move freely throughout the pastures and are provided food daily by HREC staff. Water is available on demand.

We equipped the 5 guardian dogs (2 females and 3 males) with iGotU GT-600 GPS loggers (Mobile Action Technology, Inc., Taiwan) and AX3 tri-axial accelerometers (Axivity, United Kingdom). The guardian dogs experience minimal contact with their human handlers, other than to receive food or veterinary treatment. This prevents the dogs from abandoning their sheep herds in preference for contact with people, and in this study serves to emulate the reaction wild canids might have to research equipment. The GPS devices weighed 37 g and the accelerometers weighed 11 g, well under the standard 5% body weight threshold for biologging devices^50^. GPS were set to record location every 5 seconds (i.e. 0.2 Hz), and accelerometers were set to register acceleration at 50 observations per second (i.e. 50 Hz). GPS devices were attached to collars equipped to the neck. The silastic harness developed for the domestic dogs would have been unsuitable for free-living animals because it is not durable enough. To trial a method of device attachment that is broadly applicable to wild animals, accelerometers were affixed to the dorsal-pelvic region of the dogs via a temporary epoxy adhesive (Poxipol; Akapol, Argentina) directly to this region after trimming the fur (see Supplementary Material for more details). The position of the logger on the lower back ensured that the animal was unable to reach or tamper with the device. After device attachment, we video-recorded behaviour of each dog for one hour in order to obtain observations of scent marking that could be used to train the k-NN classifier. The devices were retrieved from the dogs after between 3 and 7 days, by cutting the fur beneath the logger. The site of attachment was monitored for 3 weeks following device retrieval in order to ensure that there were no lasting deleterious effects (only some mild reddening of skin immediately after detachment, see Supplementary Information).

### K-Nearest neighbour algorithm

The KNN algorithm is an intuitive and conceptually simple supervised machine learning algorithm for classification problems^45^. The models are considered ‘supervised’ because they are trained using validated data where the class of interest in known, after which they can be applied to predict the classes of ‘new’ data (i.e. unlabelled data from unobserved animals). The algorithm has two main stages that are repeated for each unlabelled point; first the distance between new unlabelled data points and each of the labelled training points is calculated. This distance can be measured in a number of ways, but the most common is to use the Euclidean distance in the units of measurement (in our case .*g* in three dimensions corresponding to the axis of the accelerometer). The second stage involves ranking all training points by their distance to the unlabelled data, and for each point, taking a sample of nearest points. The size of this sample is determined by the hyperparameter *k*, hence the algorithm’s name *k-nearest neighbour*. The class of the unlabelled data is then determined by a majority vote of the k-nearest neighbours, i.e. whichever class is most abundant in the k sample is assigned to the unlabelled data point. For an illustrated example of the KNN we refer readers to Bidder *et al*.^45^.

It is often preferable to select a value for k that is indivisible by 2, so as to avoid a potential deadlock in the majority vote. No sure-fire method exists for determining optimum values for k (c.f.^53^), but a small k is preferred because nearer points hold more relevant information about the potential class of the new data point. This issue can also be dealt with by using a voting scheme that weights training points based on their proximity to testing points^54^. KNN has been demonstrated to be an effective method of classifying accelerometer data by behaviour type^45,55^. We used the implementations of KNN available in the scikit-learn library for Python^56^ for both the validation experiment on domestic dogs and for the deployments on the guardian dogs. We used Python because this language is optimised for large datasets and scikit-learn provides a convenient framework for cross-validation and tuning of the k hyperparameter. However, to enable researchers to utilize the methods presented in this study we provide the Python code needed in the Supplementary Information.

For the validation experiment on the domestic dogs, we adapted the procedure of Bidder *et al*.^45^. We built a training data set using a random sample of 50 raw triaxial accelerometer records from each of the scent marking classes observed (e.g. left leg raised, right leg raised, squat urination, defecation) and 500 raw triaxial accelerometer records from periods where no scent marking postures were observed (i.e. during locomotion, rolling, resting) and concatenated these in to a general ‘other’ behavioural class. We used this low proportion of available labelled data for training to emulate situations where only a few examples of each posture are observed prior to release. We then performed grid search hyperparameter tuning for *k* with 5 cross validation folds using the scikit-learn library. Finally, we used the trained model with the tuned value for k to predict the behaviour for a testing set that consisted of all occurrences of scent marking during observation, excluding those records used to train the model, and 3000 randomly selected records of ‘other’ behaviours. We randomly selected records for which ‘other’ behaviour was observed because these occurred 50–100 times more often than scent marking behaviours during the observation period, and we didn’t want the performance metric scores to be driven by ‘other’ behaviours alone. Had we not done this, it would be possible for our model to erroneously predict ‘other’ as the class for all records, and still score an accuracy that was equal to the overall proportion of ‘other’ in the data set (ca. 95% or more). As an additional component to our analysis, we also used the models trained for each dog to predict the classes of the testing data for all other dogs, trialling each training-dog testing-dog combination in a pairwise fashion. We did this in order to assess how similar in morphology a potential surrogate animal may need to be in cases where researchers aimed to study species for which captive individuals did not exist or for which a period of observation subsequent to release would be difficult.

By comparing the predicted classes of our testing set with the known classes (as determined by observation with a camera) we were able to calculate a range of performance metrics available in the scikit-learn library, namely; accuracy, precision, recall, f1 score and area under the receiver operator characteristic curve (AUC). Accuracy is the proportion of samples that were correctly classified. If $y$ is the true class and $\hat{y}$ is the predicted class, accuracy is calculated as;

1 $${\rm{accuracy}}(y,\hat{y})=\frac{1}{{n}_{{\rm{samples}}}}\,\mathop{\sum }\limits_{i=0}^{{n}_{{\rm{samples}}}}\,1({\hat{y}}_{i}={y}_{i})$$

Given that our classification problem is a multiclass (rather than binary) one, precision, recall, f1 score and AUC must be calculated for each class separately and then combined using an average weighted by prevalence of each class to give a single metric of performance. Predictions are either positive or negative for a given class (i.e. they are either 1 for the class in question or 0 and thus must be one of the other classes). These positive and negative predictions can also be true or false based on whether they matched the known classification at that time, as determined through visual observation of the dogs. Thus, for each class in the data set we obtain a set of classifications that must be one of either True Positive (TP), False Positive (FP), True Negative (TN) or False Negative (FN) values. From these we can calculate our metrics^57^ using the following;

2 $${\rm{precision}}=\frac{TP}{TP+FP}$$

3 $${\rm{recall}}=\frac{TP}{TP+FN}$$

4 $${\rm{F1}}\,{\rm{Score}}=2\ast \frac{{\rm{precision}}\ast {\rm{recall}}}{{\rm{precision}}+{\rm{recall}}}$$

Thus, precision is the proportion of positive classification that were correct, recall the proportion of true values for that class that were given a positive classification, and f1 score a harmonic mean of both precision and recall. AUC is often used to characterize the trade-off between true positive rate (i.e. recall) and the true negative rate, with a score of 1 indicating no false positives or false negatives. Random guessing would return a score of 0.5. We do not offer a detailed description of AUC or receiver operator characteristic analysis here, but we refer readers to Fawcett^58^ for additional details.

For the guardian dog experiment, we performed validation through an initial period of observation that contained at least 1 scent marking event for females and at least 1 of each left and right leg scent marks for the males. We performed manual annotation of the accelerometer data using the free software ELAN (Max Planck Institute for Psycholinguistics, Germany), using 7 general behavioural classes: walk, run, jump, stand, lie, sit and shake; and either squat or right and left leg raised scent marks depending if the subject was male or female. No defaecations were observed for the guardian dogs during the observation period. The data for the observation period was randomly split in to training and testing sets based on a 0.66/0.33 split, and the KNN fitted to the training data using *k* tuned for each dog in a manner similar for the dogs in the validation experiment. We evaluated accuracy and f1 score (calculated as above) for each subject’s KNN classifier.

We then ran a smoothing function over the predictions for both observed and unobserved periods, in order to reduce the data volume and provide a means to synchronise with less resolute GPS location data. To do this, we segmented the data in to bins of 1 second in duration and then took the modal class for each bin. The down-sampled data classifications were also compared to known observations to calculate accuracy at a 1 Hz rate, and a behaviour specific accuracy. Calculating behaviour specific accuracy ensures that scent marks are being classified correctly (which is the aim of this study), and that high accuracy scores are not being driven by more frequently expressed behaviours. All the computational scripts required for these analyses are provided in Supplementary Information, in order to encourage uptake of this approach by the research community.

### Scent marking indices and statistics

We illustrate possible uses for this novel spatial and temporal description of scent marking behaviour, obtained through application of the KNN to accelerometer data obtained concurrently with GPS location data. The concepts of home-range and territory differ in that the latter consists only of areas actively defended against competitors^59^, following Noble’s succinct definition that “territory is defended area”^60^. To illustrate this difference, we calculated a 95% minimum convex polygons (MCP)^61^ from all GPS telemetry locations and a second MCP using locations associated with scent marking activity only (as identified via KNN analysis). The calculation of 95% MCPs was conducted in the R statistical environment using the adehabitatHR package version 0.4.14^62^. We then calculate the area of overlap between MCP produced from the two data types using the ‘rgeos’ package^63^, expressing overlap as the proportion of the all-location MCPs that are also included in the scent mark only MCPs.

To examine revisits and overmarks, we used functions in the ‘rgeos’ R package to detect events in which dogs marked within 10 m of a previous scent mark, and calculated the time between visits based on the timestamp of the GPS telemetry data. We chose a 10 m buffer distance for these events because the accuracy of the GPS data is ca. 10 m. Canid species are known to detect scents within this distance^64–66^, and often overmark when they do^29,67,68^. Thus, if a scent-mark is observed within 10 m of a competitor’s, there is a high probability that the animal under observation will have attempted to place their scent-mark as close to it as possible. Again, the purpose of the present study is to illustrate possible use-cases for this novel information type, and here we avoid generalizing too much from the behaviour we observe.

If the area delineated by scent marks is taken as its territory, then excursions from this area may constitute extra-territorial forays^69–71^. To describe this behaviour type, we used ‘rgeos’ and ‘raster’^72^ R packages to count and time each extra-territorial excursion. We took the first location detected outside of the scent-mark delineated 95% MCP as the start location and time, with the last location prior to re-entry taken as the end point and time. We separated extra-territorial forays in to those over 10 minutes in duration and those under, although this threshold was selected arbitrarily in our example use-case. We report the mean duration, total duration and percent of total deployment time spent conducting extra-territorial forays for our guardian dogs in the results.

All of our example guardian dog use-cases are visualized using the ggmap R package^73^, with Google’s ‘terrain’ basemap (characterizing slope) used in order to provide spatial context.

## Results

### Domestic dog validation

Of the 62 dogs equipped and tested, only 31 could be submitted to KNN analysis whilst the others were excluded for the following reasons: not enough scent marks conducted during the study period (n = 20), video file corruption (n = 2), accelerometer failure (n = 2), harness rejection (n = 2) and a data labelling issue where dogs’ files were lost (n = 5). The results for the remaining 31 dogs submitted to analysis are summarized in Table 1. These results suggest that using a k of 3, as determined through 5-fold cross validation, provides adequate performance in all of the metrics used.

**Table 1:** Performance metrics by sex observed during validation experiment using domestic dogs.

Sex	N	Modal K	Accuracy	Precision	Recall	F1 Score	AUC
F	12	3	0.926	0.931	0.928	0.928	0.929
M	19	3	0.927	0.929	0.930	0.928	0.924

**Figure 1:**
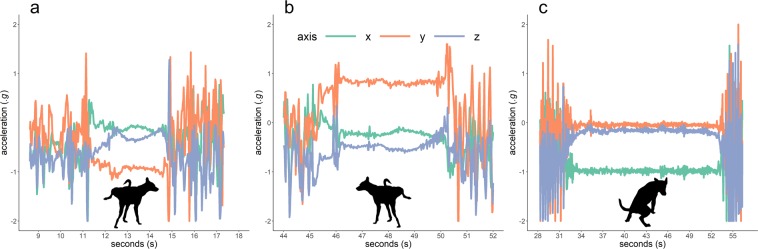
Accelerometer signals for left leg (**a**), right leg (**b**) and squat (**c**) postures. Dog silhouette illustrations provided by Zoe Beba.

The posture adopted when placing scent marks differed between male and females, as males used the archetypal canine ‘leg-up’ posture whilst females predominantly used the ‘squat’ posture. We wished to ensure that the KNN classifier was able to differentiate between female ‘squat’ posture urinations and defaecations. In consideration of this, we the performance metrics for both males and females separately in Table 1, where there is no discernible difference in accuracy between males and females. Inspection of the signatures produced by the accelerometers provides some insight in to why the KNN classifier was able to perform so well. Figure 1 illustrates the signatures produced by left leg (panel a), right leg (panel b) and a squat to defaecate (panel c). Left and right leg up postures produce polarization in the lateral y axis (i.e. sway), providing values of approximately −1 g and 1 g respectively. The dogs hold these static postures for a few seconds, producing conspicuous signals that can be detected by the KNN classifier. A similar signature is observed when the animal is in the squat posture, but instead the signal is detected along the x axis (i.e. surge) axis, producing a value of −1 g, indicating that this axis is directed toward the centre of earth’s gravity.

**Figure 2:**
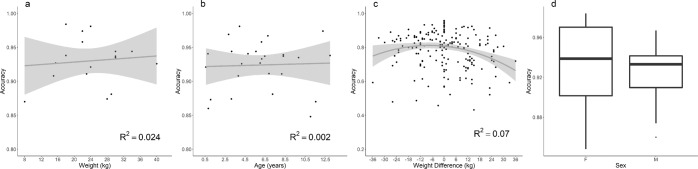
Accuracy by weight (**a**), age (**b**) and sex (**c**), illustrating that these factors have no influence on our ability to detect scent marking behaviour.

An alternative three-dimensional view of this same data is given in the Supplementary Information (Fig. S4) to provide an intuitive illustration of how the KNN classifies new accelerometer data according to its proximity to training data. A specific aim of this study was to develop a method that was broadly applicable across canid species of varying morphology. Classification accuracy was unaffected by the weight, age or sex of the animals used in our validation study (Fig. 2), suggesting that the method does not perform any worse on very small or very large dogs. This leads us to conclude that the method does not suffer any reduction in efficacy due to morphology, and should be broadly applicable. In certain cases researchers may wish to train the model using a surrogate individual before detection of scent marking postures in another. To emulate this scenario, we used models trained on each male dog to predict the scent marking activity in all other males. Figure 2(c) details the results of this pairwise analysis. We found no relationship between the weight difference of dogs and model performance when models are trained on one dog and applied to another for prediction. Some dog-pairs produced comparable performance to when training and testing was performed on the same individual, whilst others dog-pairs performed poorly. For a detailed table of results for each surrogate, see the Supplementary Information (Table S1).

### Guardian dog deployments

The epoxy adhesive failed prematurely for 2 of the guardian dogs equipped with accelerometer and GPS loggers, so complete datasets were available for 3 guardian dogs. One of the dogs was moved by handlers to protect livestock in a second area mid-way through the deployment, so we separated the periods in each pasture for analysis. An initial period of validation was performed on each dog, and confirmed high classification accuracy (Table 2). Classification of accelerometer data for the unobserved period revealed 61 individual scent mark events across 218.76 dog-hours monitored. For a summary of scent marking rates and total deployment duration for each dog, see Table 3.

**Table 2:** Accuracy information for each guardian dog.

Dog	Sex	K	Overall Accuracy	Overall F1 Score	Left Detections	Right Detections	Squat Detections
Dog1	F	7	98.4%	96.1%	—	—	7/7
Dog2	F	21	92%	91.7%	—	—	7/7
Dog3	M	15	90.3%	88.8%	3/4	4/6	—

Scent mark specific detection rates are for the proportion of total seconds in each posture that were detected.

**Table 3:** Summary of scent mark count for each guardian dog.

Dog	Sex	N Scent Marks	Deployment Duration (hours)	Rate (SM/hour)
Dog1-A	F	18	19.72	0.91
Dog1-B	F	12	29.13	0.41
Dog2	F	21	46.89	0.45
Dog3	M	9	123.11	0.07

**Figure 3:**
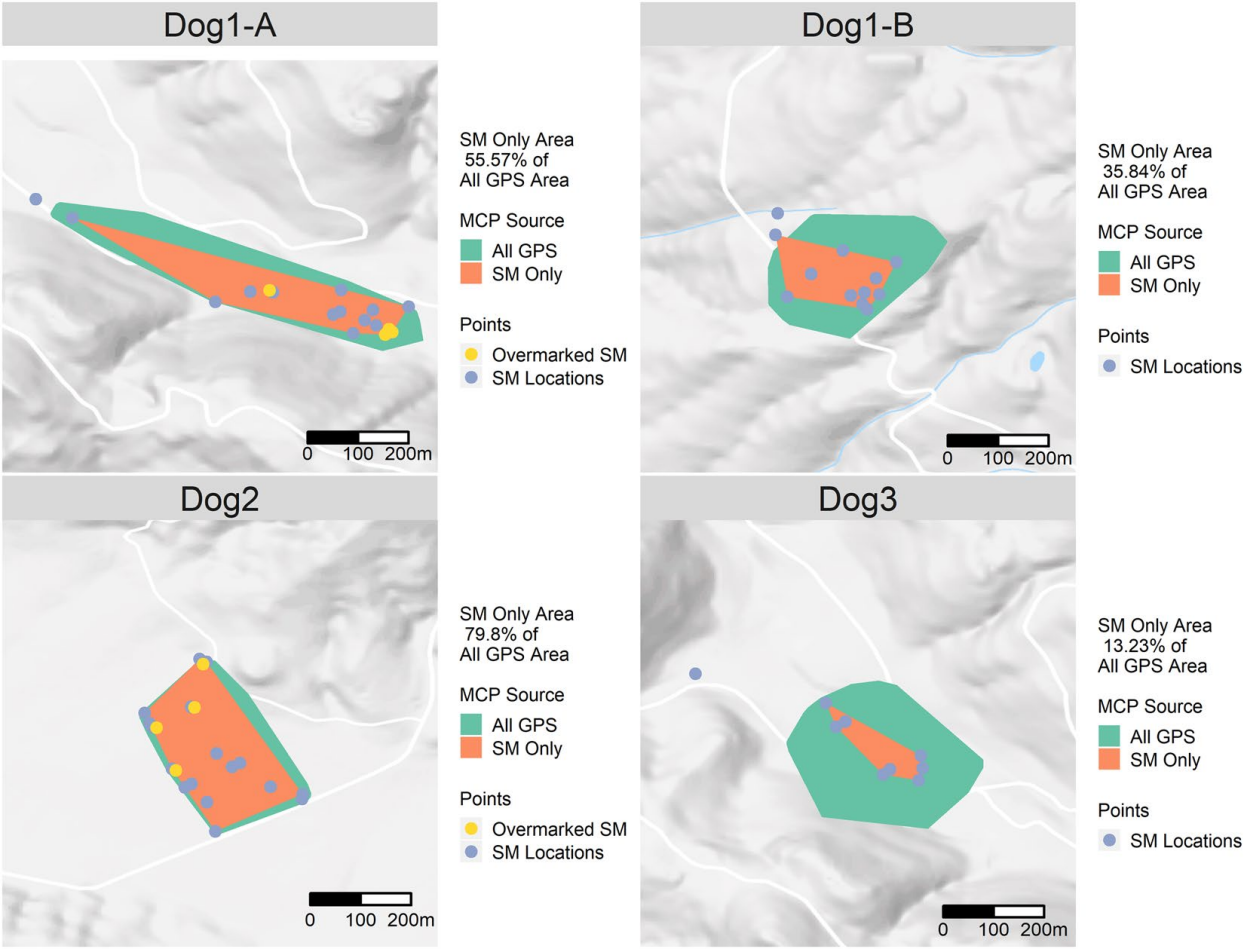
95% MCP derived from GPS Locations and Scent Marks. Overmarked locations shown in yellow. Google terrain base map included for context.

**Figure 4:**
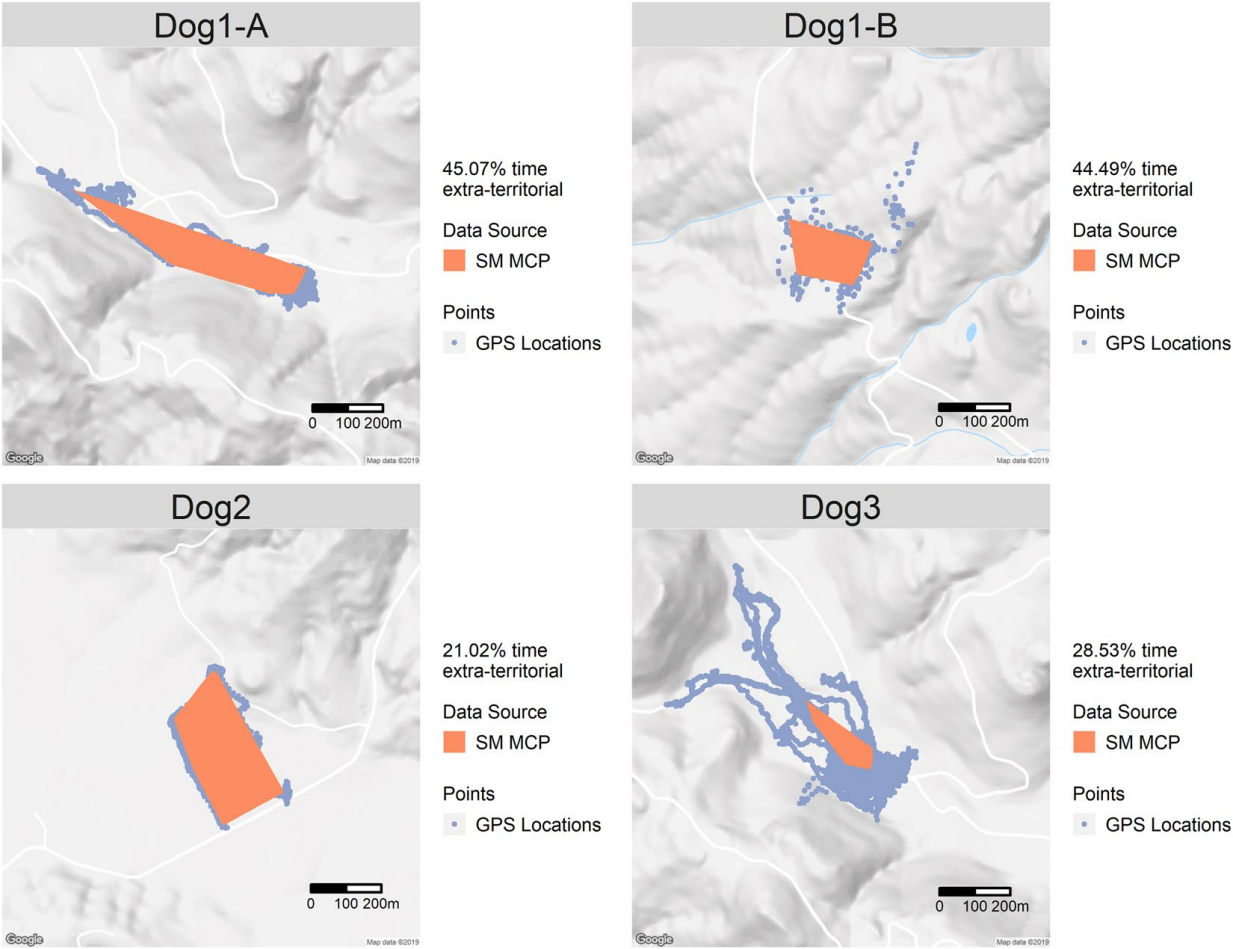
Scent Mark derived MCP with extra-territorial locations shown. Google terrain base-map shown for context.

The spatial distribution of scent marks for each dog is shown in Fig. 3, along with a comparison between home-ranges (as calculated by a 95% MCP drawn around all GPS locations) and territory (derived from a 95% MCP using scent-mark locations). The home-range area was larger for all individuals, with the mean proportion of home-range overlap with territory at 46.1%. We were able to detect revisit overmarks on 4 instances each for both Dog1-A and Dog2. Mean revisit time for these were 5.3 hours and 12.3 hours for Dog1-A and Dog2 respectively. We designated periods spent outside the territory MCP as extra-territorial movements. Figure 4 shows these extra-territorial excursions for each guardian dog. The frequency and duration of excursions varied between the guardian dogs, and partly depended on whether they placed scent marks on the periphery of their home-range. Table 4 summarises the number and duration of these excursions, revealing an interesting pattern of short, frequent excursions and infrequent long excursions that probably were a result of each dog’s territory size, resting site locations and responses to sheep.

**Table 4:** Details on the number and duration of extra-territorial forays by each of the guardian dogs.

Dog	N excursions under 10 min	N excursions over 10 min	Mean, Std dev excursion duration (min)	Total extra-territorial time (min)	Percent extra-territorial	Excursion frequency (N per min)
Dog1-A	67	7	7.9 (±31.1)	588.05	45.1%	0.06
Dog1-B	21	20	23.9 (±50.4)	978.42	44.5%	0.02
Dog2	419	7	1.4 (±5.4)	635.67	21%	0.05
Dog3	383	54	5.4 (±14.4)	2359.74	28.5%	0.14

## Discussion

The novel method we present here provides a means to study scent marking in the wild by individually-identifiable wild animals without the need for direct observation. This new method may provide novel insights into this important yet understudied ecological phenomenon, one that has profound consequences for individual fitness, social organisation, and community ecology.

### Technique validation and accuracy

Our techniques for detecting scent marking proved effective, with accuracy scores above 90% for the validation trial conducted with domestic dogs (see Table 1). These high accuracy scores are comparable to those of studies of other taxa in which accelerometers monitor behaviour^41,55,74^. In fact, accuracy scores for this application of KNN were higher than those for other species in previous studies^37,45,55,75^. The high performance in this application is probably due to the marked differences in posture (and thus device orientation) adopted when canids scent mark. In Fig. 1, conspicuous differences in the accelerometer signal are visible between left-leg, right-leg, and squat postures. The fact that these postures are typically held for a few seconds with little dynamic movement probably aids detection, and offers significant signal contrast between these and other postures (see Supplementary Information for further illustration in a 3-dimensional representation). For females, we identified urination and defaecation postures separately, because wild canids may use either for olfactory communication (e.g.^76^), and because we expected less contrast between these postures compared to the left-leg and right-leg postures observed predominantly in males.

Our aim was to develop a method that is broadly applicable to both males and females across canid species. There was no reduction in accuracy between males and females (Table 1) and the KNN algorithm detects the different squat and raised-leg postures equally well. For females, the method is robust to small differences between urination and defaecation squat postures, which is germane for species that use faecal olfactory signals^76,77^. We validated the method with breeds of a range of sizes (2.6 kg to 40 kg) that emulate those observed for most adult canids, (0.98 kg to 31.7 kg^48^), to ensure the method works equally well on canids of all sizes. Accuracy was not affected by weight (a proxy of animal morphological size) or age (Fig. 2), providing evidence that the method is broadly applicable across the various morphologies of canid species (c.f.^74^).

### Considerations for application

While accelerometers represent a promising methodology for studying scent marking behaviour, there remain limitations to its application. Like all supervised machine-learning methods, the KNN requires validated training data, typically obtained during an observation period or by using captive individuals^45^. This may not always be possible for species that are difficult to observe or for which few captive individuals are available.

There are two possible approaches to overcome this issue. The first involves using a surrogate species to obtain a validated training set. This approach has been demonstrated in canids, with little loss of accuracy when classifiers are used on morphologically similar species^74^. In our study, comparison between the accelerometer signatures produced by the domestic dogs revealed a generalisable pattern that could be applied across individuals (Fig. 1). We performed pairwise application of models trained on one dog to predict the behaviour of another (Fig. 2c), which revealed no discernible relationship between the difference in weight (a proxy for body size) and model performance, with the caveat that models still performed best when the same animal was used for both training and testing. However, these results do raise the possibility of using dogs as surrogates for wild canids. Domestic dog breeds could also serve as convenient proxies for many wild canids because of their immense morphological variation^78^.

The second approach for training a classifier when validation data is difficult to obtain, is through manual annotation of data from wild deployment. Manual annotation can be conducted following the principles set out by Shepard *et al*.^79^, and does not necessitate an observational period^45,80^. Researchers may also use thresholding^81^ or Boolean pattern matching^82^ to quickly identify the conspicuous signal produced during scent marking (Fig. 1), and build a training set after visual verification. Unsupervised methods, such as k-means clustering, may also be used^83^. In developing this method, we found that scent marking results in a distinct, easily-distinguishable pattern that would be straightforward to identify during manual annotation.

When deploying accelerometers to study scent marks, device placement is critical. Scent marking must be associated with a predictable body posture that can be detected through changes in device orientation. Typically, it’s impossible for the accelerometer to detect this pelvic tilt when mounted to collars around the neck, and so this necessitates mounting an additional accelerometer on the pelvis (preliminary trials showed no discernible signal from collar mounted accelerometers). Complementary GPS data is still necessary to obtain the locations of scent marks however, and so the devices must be used in concert. Also, the spatial accuracy of scent mark locations subject to the errors of GPS locations, which vary with habitat, potentially introducing bias^84^.

Device retrieval remains a barrier to widespread uptake of fine-scale accelerometery in general, since technology does not yet exist to facilitate the remote transmission of the gigabytes of data these devices produce^85^. Developing methods for remote download of accelerometer data represents an important frontier for the biologging community. For now, the use of accelerometers is best suited to species that have high site fidelity, including territorial or denning species^86^ or those that return to predictable haul-out sites or colonies^87^. If animals cannot be recaptured, the device must be equipped with a transmitter such as VHF, so that the device can be recovered once detached (e.g.^88–90^). Published research using the same adhesive attachment mechanism used here demonstrated successful attachment for ca. 30 days^47^. Provided a VHF transponder is used, a ca. 30-day attachment period should limit the animal from travelling too far and provide a manageable search area for locating the device, especially if animal movement is concentrated in territories or around den sites.

### Research applications

An additional aim of this study was to provide a tractable illustration of the types of data this technique provides. Here, we demonstrate how existing concepts in movement ecology can be extended to open new avenues for research on scent marking, an important form of communication. We provide an example use-case through application of the technique on guardian dogs, tying the information gained to questions pertinent to animal ecology.

### Territorial defence

Home-range and territory differ in that only territories are actively defended against intrusion^59^. As a result, territories typically constitute only a portion of the larger home-range in which an animal is observed. Although there are many sophisticated methods to estimate animal home-ranges^91^, establishing defended territory within that range is difficult. Figure 3 provides an illustration of the distinction between home-range and territory for our guardian dogs, through calculation of a MCP derived from GPS location data and another derived from scent marking locations only. While this only serves as an example using this novel approach, we observed the proportion of the total home-range that is demarked by defensive scent marks ranges from ca. 13.23% to 79.8%. Home-ranges derived from GPS data alone don’t necessarily provide information on what features in the environment are of utility to animals, they merely describe the probability of locating an animal at any given location^92^. Animals are territorial when critical resources are in short supply and limit population growth^93^. As such, conditions within their territories should make better predictors of productivity (i.e. breeding success of pairs and packs) and subsequently population change over time, than conditions measured from their larger home-ranges. Thus, the ability to delineate territories within home-ranges is important. The method presented here provides a workable means to establish which areas are being actively defended through scent-marking.

The rate of scent marking at the territory boundary may provide insight into the territorial pressure exerted by conspecifics^22^. The spatial positioning of scent marks within the territory may also indicate their function. Scent marks on the periphery of a territory may pertain to territorial defence^26,94^, whilst those towards the interior may fulfil a variety of functions pertaining to intra-social group communication and social organisation^11,29^. Although the nuances of these interior scent marks are less understood, with the techniques introduced here, it is now possible to determine which scent marks are near the territory periphery and which the interior (Fig. 3). If the guardian dogs were actively defending territory, it is likely that we would observe greater scent marking activity on territorial boundaries adjacent to competitor’s territories, as has been observed in grey wolves^95^. An added benefit of our method is that it is possible to determine whether scent marks are placed using raised leg or squat urinations, which may also communicate whether scent marks are intended for territorial defence^29,96^.

### Extra-territorial forays

Once the territorial boundaries have been established, the number, length and frequency of extra-territorial forays can be assessed. Figure 4 shows the locations obtained from each of the guardian dogs outside of their territory, and the number and duration of extra-territorial forays is described in Table 5. The information obtained with our approach could provide important insights on territorial behaviour of guardian dogs and their interaction with other species. For instance, while some researchers have stated that a guardian dog’s efficiency in preventing predation depends on the direct effect generated by presence^97^, others have shown that guardian dogs maintain territories and chemically respond to potential predators^98^. Our approach allows us to measure scent marking continuously through time and space and assess when and where guardian dogs display territorial behaviours and extra-territorial movements. With additional information on livestock movement patterns, we might also assess whether guardian dogs perform extra-territorial forays to corral wandering livestock, challenge predators, or abandon their wards. This information is particularly valuable to practitioners hoping to use guardian dogs in lieu of lethal predator control. For other canid species, extra-territorial forays are important during dispersal, periods of food shortage, and play a role in the social dynamics between individuals that occupy neighbouring territories^12,15,16^.

### Temporal dynamics of scent marking

Scent marks collected from snow reveal only vague information on how many animals have previously visited it, and almost none on the time between visits. In addition to recording location, our method describes scent mark distribution in time, providing an assessment of the time between over marking events. The incidence of self-over-marking was detected for two of the guardian dogs (Fig. 3), although none overlapped in time and space concurrently to assess over-marking between individuals. Self-overmarking may be necessary for wild animals to maintain scent-mark efficacy^4^, and to establish well defined and stable territories^13^. By revisiting well-placed scent marks, individuals maintain their olfactory signals and convey additional information about their revisitation rate and thus the likelihood that intruders will encounter the territory holder^11^. The revisitation rate may also convey to researchers the resource value of that location^99^. This contrasts with utilization distributions, which are likely to under represent important sites that are used infrequently or briefly^92^. Over-marking is an important social process, implicated in pair-bonding^100,101^, as an agonistic response to territorial encroachment^32^, or for amplification of within-group scent marks to convey additional information on pack size and composition to would-be-intruders (e.g.^34^). The novel bio-logging approach proposed here provides a record of scent marking activity over a period long enough to assess revisitation and over-marking activities for the first time.

## Conclusion

We established how scent marking behaviour in canids can be detected using accelerometers and machine-learning techniques. This is a meaningful first step in addressing the information gap that exists for this important form of olfactory communication. Thus far, lack of tractable methods has prohibited progress in this topic. However, biologging technologies such as accelerometers have already greatly advanced our understanding of difficult to observe species and phenomena^102^. When researchers can easily leverage these technologies, a rapid proliferation of applications and insights has often followed^103,104^. Now researchers of canid behavioural ecology and community ecology are empowered to harness this new information source and combine it with analytical advances in the field of movement ecology. By doing so, researchers can shed new light on how scent marking functions with respect to external factors (e.g. other organisms, environment) and internal state (e.g. endocrine function, nutritional status). Canid species, many of which are of conservation concern^105–107^, the focus of intense management efforts^108^ and key to the proper functioning of ecosystems^109,110^, rely primarily on scent for communication. Olfactory messages are key to their social cohesion, spatial organisation and competitions for resources. However, as a species that depends so heavily on vision, we humans are unequipped to detect olfactory signals, until now limiting research on this topic. We hope with this new technique, ecologists will now be able to better observe these invisible conversations, leading to a greater understanding of a sensory world so different from our own.

## Supplementary information

Supplementary Information.

## References

[1] Hornocker MG (1969). Winter Territoriality in Mountain Lions. J. Wildl. Manage..

[2] Gese EM (2001). Territorial defense by coyotes (Canis latrans) in Yellowstone National Park, Wyoming: who, how, where, when, and why. Can. J. Zool..

[3] Marsden HM, Bronson FH (1964). Estrous synchrony in mice: Alteration by exposure to male urine. Science (80-.)..

[4] Roberts SC, Dunbar RIM (2000). Female territoriality and the function of scent-marking in a monogamous antelope (Oreotragus oreotragus). Behav. Ecol. Sociobiol..

[5] Michael RP, Keverne EB (1968). Pheromones in the communication of sexual status in primates. Nature.

[6] Ralls K (1971). Mammalian scent marking. Science.

[7] Zala SM, Potts WK, Penn DJ (2004). Scent-marking displays provide honest signals of health and infection. Behav. Ecol..

[8] Russell BG, Banks PB (2007). Do Australian small mammals respond to native and introduced predator odours?. Austral Ecol..

[9] Anisko, J. J. Chapter 14: Communication by Chemical Signals in Canidae. In *Mammalian olfaction, reproductive processes, and behavior* (ed. Doty, R. L.) 283–292 (Academic Press, 1976).

[10] Gosling, L. M. Economic consequences of scent marking in mammalian territoriality. In *Chemical Signals in Vertebrates 4* (eds. Duvall, D., Muller-Schwarze, D. & Silberstein, R. M.) 385–395 (Plenum Press, 1986).

[11] Mech, L. D. *The wolf: ecology and behaviour of an endangered species*. (1970).

[12] Soulsbury CD, Iossa G, Baker PJ, White PCL, Harris S (2011). Behavioral and spatial analysis of extraterritorial movements in red foxes (Vulpes vulpes). J. Mammal..

[13] Briscoe B, Lewis MA, Parrish SE (2002). Home Range Formation in Wolves Due to Scent Marking. Bull. Math. Biol..

[14] Van Ballenberghe V (1983). Extraterritorial movements and dispersal of wolves in southcentral alaska. J. Mammal..

[15] Messier F (1985). Solitary living and extraterritorial movements of wolves in relation to social status and prey abundance. Can. J. Zool..

[16] Mech LD (1977). Productivity, Mortality, and Population Trends of Wolves in Northeastern Minnesota. J. Mammal..

[17] Lewis MA, Moorcroft P (2001). ESS Analysis of Mechanistic Models for Territoriality: the Value of Scent Marks in Spatial Resource Partitioning. J. Theor. Biol..

[18] Moorcroft PR, Barnett A (2008). Mechanistic home range models and resource selection analysis: A reconciliation and unification. Ecology.

[19] Moorcroft APR, Lewis MA, Crabtree RL, Ecology S, Jul N (2012). Home Range Analysis Using a Mechanistic Home Range. America (NY)..

[20] Parsons MH (2018). Biologically meaningful scents: a framework for understanding predator-prey research across disciplines. Biol. Rev..

[21] Muller‐Schwarze, D. *Chemical Ecology of Vertebrates*. (Cambridge University Press, 2006).

[22] Pal S (2003). Urine marking by free-ranging dogs (Canis familiaris) in relation to sex, season, place and posture. Appl. Anim. Behav. Sci..

[23] Trenberth KE (1998). Atmospheric moisture residence times and cycling: Implications for rainfall rates and climate change. Clim. Change.

[24] Ghaskadbi P, Habib B, Qureshi Q (2016). A whistle in the woods: an ethogram and activity budget for the dhole in central India. J. Mammal..

[25] Soulsbury CD, Fawcett JK (2015). Ontogenic patterns of scent marking in red foxes, Vulpes vulpes (Carnivora: Canidae). Folia Zool..

[26] Allen JJ, Bekoff M, Crabtree RL (1999). An Observational Study of Coyote (Canis latrans) Scent-marking and Territoriality in Yellowstone National Park. Ethology.

[27] Burghardt GM (2012). Perspectives - Minimizing Observer Bias in Behavioral Studies: A Review and Recommendations. Ethology.

[28] Tuyttens FAM (2014). Observer bias in animal behaviour research: can we believe what we score, if we score what we believe?. Anim. Behav..

[29] Jordan NR, Golabek KA, Apps PJ, Gilfillan GD, McNutt JW (2013). Scent-Mark Identification and Scent-Marking Behaviour in African Wild Dogs (Lycaon pictus). Ethology.

[30] Barrette C, Messier F (1980). Scent-marking in free-ranging coyotes, Canis latrans. Anim. Behav..

[31] Henry, J. D. The Urine Marking Behavior and Movement Patterns of Red Foxes (Vulpes Vulpes) During a Breeding and Post-Breeding Period. In *Chemical Signals* 11–27, 10.1007/978-1-4684-1027-3_2 (Springer US, 1980).

[32] Paquet PC (1991). Scent-marking behavior of sympatric wolves (Canis lupus) and coyotes (C. latrans) in Riding Mountain National Park. Can. J. Zool..

[33] Rothman RJ, Mech LDD (1979). Scent-marking in lone wolves and newly formed pairs. Anim. Behav..

[34] Sillero-Zubiri C, Macdonald DW (1998). Scent-marking and territorial behaviour of Ethiopian wolves Canis simensis. J. Zool..

[35] Cooke SJ (2004). Biotelemetry: a mechanistic approach to ecology. Trends Ecol. Evol..

[36] Støen OG (2015). Physiological evidence for a human-induced landscape of fear in brown bears (Ursus arctos). Physiol. Behav..

[37] Williams HJ, Shepard ELC, Duriez O, Lambertucci SA (2015). Can accelerometry be used to distinguish between flight types in soaring birds?. Anim. Biotelemetry.

[38] Watanuki Y, Mehlum F, Takahashi A (2001). Water temperature sampling by foraging Brunnich’s Guillemots with bird-borne data loggers. J. Avian Biol..

[39] Sala JE, Pisoni JP, Quintana F (2017). Three-dimensional temperature fields of the North Patagonian Sea recorded by Magellanic penguins as biological sampling platforms. Estuar. Coast. Shelf Sci..

[40] Brown DD, Kays R, Wikelski M, Wilson RP, Klimley AP (2013). Observing the unwatchable through acceleration logging of animal behavior. Anim. Biotelemetry.

[41] Nathan R (2012). Using tri-axial acceleration data to identify behavioural modes of free-ranging animals: general concepts and tools illustrated for griffon vultures. J. Exp. Biol..

[42] Ladds MA (2017). Super machine learning: Improving accuracy and reducing variance of behaviour classification from accelerometry. Anim. Biotelemetry.

[43] Gao L, Campbell H, Bidder OR, Hunter J (2013). A Web-based semantic tagging and activity recognition system for species’ accelerometry data. Ecol. Inform..

[44] Jeantet, L. *et al*. Combined use of two supervised learning algorithms to model sea turtle behaviours from tri-axial acceleration data. *J. Exp. Biol*. **221**, jeb.177378 (2018). 10.1242/jeb.177378 29661804

[45] Bidder OR (2014). Love thy Neighbour: Automatic animal behavioural classification of acceleration data using the K-Nearest Neighbour algorithm. PLoS One.

[46] Peters, R. & Mech, L. D. Scent-Marking in Wolves. In *Wolf and Man* 133–147, 10.1016/B978-0-12-319250-9.50015-3 (Academic Press, 1978).

[47] Lush L (2018). Classification of sheep urination events using accelerometers to aid improved measurements of livestock contributions to nitrous oxide emissions. Comput. Electron. Agric..

[48] Jones KE (2009). PanTHERIA: a species-level database of life history, ecology, and geography of extant and recently extinct mammals. Ecology.

[49] Portugal SJ, White CR (2018). Miniaturization of biologgers is not alleviating the 5% rule. Methods Ecol. Evol..

[50] Kenward RE (2002). A manual for wildlife radio tagging, 2nd edition. Anim. Conserv..

[51] Sacks BN, Jaeger MM, Neale JCC, McCullough DR (1999). Territoriality and Breeding Status of Coyotes Relative to Sheep Predation. J. Wildl. Manage..

[52] van Bommel L, Johnson CN (2014). How guardian dogs protect livestock from predators: territorial enforcement by Maremma sheepdogs. Wildl. Res..

[53] Hassanat, A. B., Abbadi, M. A., Altarawneh, G. A. & Alhasanat, A. A. Solving the Problem of the K Parameter in the KNN Classifier Using an Ensemble Learning Approach. (2014).

[54] Liu, W. & Chawla, S. Class Confidence Weighted kNN Algorithms for Imbalanced Data Sets. In 345–356, 10.1007/978-3-642-20847-8_29 (Springer, Berlin, Heidelberg, 2011).

[55] McClune DW, Marks NJ, Delahay RJ, Montgomery WI, Scantlebury DM (2015). Behaviour-time budget and functional habitat use of a free-ranging European badger(Meles meles). Anim. Biotelemetry.

[56] Pedregosa F (2011). {scikit-learn}: Machine learning in {P}ython. J. Mach. Learn. Res..

[57] Powers DMW (2011). Evaluation: From Precision, Recall and F-Measure to ROC, Informedness, Markedness & Correlation. J. Mach. Learn. Technol..

[58] Fawcett T (2005). An introduction to ROC analysis. Pattern Recognit. Lett..

[59] Powell, R. A. Animal home ranges and territories and home range estimators. In *Research Techniques in Animal Ecology: Controversies and Consequences* (eds. Pearl, M. C., Boitani, L. & Fuller, T.) 65–110 (Columbia University Press, 2000).

[60] Noble GK (1939). The Rôle of Dominance in the Social Life of Birds. Auk.

[61] Laver PN, Kelly MJ (2008). A Critical Review of Home Range Studies. J. Wildl. Manage..

[62] Calenge C (2006). The package “adehabitat” for the R software: A tool for the analysis of space and habitat use by animals. Ecol. Modell..

[63] Bivand, R., Rundel, C., Pebesma, E. & Hufthammer, K. O. Interface to Geometry Engine - Open Source (GEOS): Package ‘rgeos’. *R Documentation* (2016).

[64] Kunz TH (2007). Assessing Impacts of Wind-Energy Development on Nocturnally Active Birds and Bats: A Guidance Document. J. Wildl. Manage..

[65] Smith DA (2003). Detection and accuracy rates of dogs trained to find scats of San Joaquin kit foxes (Vulpes macrotis mutica). Anim. Conserv..

[66] Wasser SK (2004). Scat detection dogs in wildlife research and management: application to grizzly and black bears in the Yellowhead Ecosystem, Alberta, Canada. Can. J. Zool..

[67] Bekoff M (2001). Observations of scent-marking and discriminating self from others by a domestic dog (Canis familiaris): tales of displaced yellow snow. Behav. Processes.

[68] Bowen WD, Cowan IM (1980). Scent marking in coyotes. Can. J. Zool..

[69] Young AJ, Carlson AA, Clutton-Brock T (2005). Trade-offs between extraterritorial prospecting and helping in a cooperative mammal. Anim. Behav..

[70] Creel, S. & Creel, N. *The African Wild Dog: Behavior, Ecology, and Conservation - Scott Creel, Nancy Marusha Creel - Google Books*. (Princeton University Press, 2002).

[71] Sillero-Zubiri C, Gottelli D, Macdonald DW (1996). Male philopatry, extra-pack copulations and inbreeding avoidance in Ethiopian wolves (Canis simensis). Behav. Ecol. Sociobiol..

[72] Hijmans, R. J. & van Etten, J. raster: Geographic analysis and modeling with raster data. (2012).

[73] Kahle D, Wickham H (2013). ggmap: Spatial Visualization with ggplot2. R J..

[74] Campbell HA, Gao L, Bidder OR, Hunter J, Franklin CE (2013). Creating a behavioural classification module for acceleration data: using a captive surrogate for difficult to observe species. J. Exp. Biol..

[75] Resheff YS, Rotics S, Harel R, Spiegel O, Nathan R (2014). AcceleRater: a web application for supervised learning of behavioral modes from acceleration measurements. Mov. Ecol..

[76] Vilà C, Urios V, Castroviejo J (1994). Use of faeces for scent marking in Iberian wolves (Canis lupus). Can. J. Zool..

[77] Barja I (2009). Decision making in plant selection during the faecal-marking behaviour of wild wolves. Anim. Behav..

[78] Boyko AR (2010). A simple genetic architecture underlies morphological variation in dogs. PLoS Biol..

[79] Shepard ELC (2008). Identification of animal movement patterns using tri-axial accelerometry. Endang Species Res.

[80] Tanha J (2012). Multiclass semi-supervised learning for animal behavior recognition from accelerometer data. in. Proceedings - International Conference on Tools with Artificial Intelligence, ICTAI.

[81] Moreau M, Siebert S, Buerkert A, Schlecht E (2009). Use of a tri-axial accelerometer for automated recording and classification of goats grazing behaviour. Appl. Anim. Behav. Sci..

[82] Wilson RP (2018). Give the machine a hand: A Boolean time-based decision-tree template for rapidly finding animal behaviours in multisensor data. Methods Ecol. Evol..

[83] Sakamoto K (2009). Can ethograms be automatically generated using body acceleration data from free-ranging birds?. PLoS One.

[84] Heard DC, Ciarniello LM, Seip DR (2008). Grizzly bear behavior and global positioning system collar fix rates. J. Wildl. Manage..

[85] Ropert-Coudert Y, Wilson RP (2005). Trends and perspectives in animal-attached remote sensing. Front. Ecol. Environ..

[86] Mahoney PJ, Young JK (2017). Uncovering behavioural states from animal activity and site fidelity patterns. Methods Ecol. Evol..

[87] Guinet C (2014). Southern elephant seal foraging success in relation to temperature and light conditions: insight into prey distribution. Mar. Ecol. Prog. Ser..

[88] Hays GC (2015). New insights: Animal-borne cameras and accelerometers reveal the secret lives of cryptic species. J. Anim. Ecol..

[89] Whitmore BM, White CF, Gleiss AC, Whitney NM (2016). A float-release package for recovering data-loggers from wild sharks. J. Exp. Mar. Bio. Ecol..

[90] Graf PM, Mayer M, Zedrosser A, Hackländer K, Rosell F (2016). Territory size and age explain movement patterns in the Eurasian beaver. Mamm. Biol..

[91] Fieberg J, Börger L (2012). Could you please phrase “home range” as a question?. J. Mammal..

[92] Kie JG (2010). The home-range concept: are traditional estimators still relevant with modern telemetry technology. Proceeding R. Soc. Biol. Sci..

[93] Brown JL (1969). Territorial behavior and population regulation in birds. The Wilson Bullitin.

[94] Wells MC, Bekoff M (1981). An observational study of scent-marking in coyotes. Canis latrans..

[95] Zub K (2003). Wolf pack territory marking in the Białowieża Primeval Forest (Poland). Behaviour.

[96] Cafazzo S, Natoli E, Valsecchi P (2012). Scent-Marking Behaviour in a Pack of Free-Ranging Domestic Dogs. Ethology.

[97] Allen LR, Stewart-Moore N, Byrne D, Allen BL (2017). Guardian dogs protect sheep by guarding sheep, not by establishing territories and excluding predators. Anim. Prod. Sci..

[98] van Bommel L, Johnson CN (2017). Olfactory communication to protect livestock: dingo response to urine marks of livestock guardian dogs. Aust. Mammal..

[99] Barja I, List R (2014). The Role of Spatial Distribution of Faeces in Coyote Scent Marking Behaviour. Polish J. Ecol..

[100] Apps P, Mmualefe L, McNutt JW (2012). Identification of Volatiles from the Secretions and Excretions of African Wild Dogs (Lycaon pictus). J. Chem. Ecol..

[101] Jordan NR, Apps PJ, Golabek KA, McNutt JW (2014). Top marks from top dogs: tandem marking and pair bond advertisement in African wild dogs. Anim. Behav..

[102] Wilmers CC (2015). The golden age of bio-logging: how animal-borne sensors are advancing the frontiers of ecology. Ecology.

[103] Rutz C, Hays GC (2009). New frontiers in biologging science. Biol. Lett..

[104] Cooke SJ (2008). Biotelemetry and biologging in endangered species research and animal conservation: relevance to regional, national, and IUCN Red List threat assessments. Endanger. Species Res..

[105] Gottelli D, Sillero-Zubiri C (1992). The Ethopian wolf - an endangered endemic canid. Oryx.

[106] Garcia-Moreno J, Matocq MD, Roy MS, Geffen E, Wayne RK (1996). Relationships and Genetic Purity of the Endangered Mexican Wolf Based on Analysis of Microsatellite Loci. Conserv. Biol..

[107] Ortega J, Franco MDR, Adams BA, Ralls K, Maldonado JE (2004). A reliable, non-invasive method for sex determination in the endangered San Joaquin kit fox (Vulpes macrotis mutica) and other canids. Conserv. Genet..

[108] Harding EK, Doak DF, Albertson D (2002). Evaluating the effectiveness of predator control: the non-native red fox as a case study. Conserv. Biol..

[109] Mannise N, Trovati RG, Duarte JMB, Maldonado JE, Gonzalez S (2001). Using non–invasive genetic techniques to assist in maned wolf conservation in a remnant fragment of the Brazilian Cerrado. Anim. Biodivers. Conserv..

[110] Wilmers CC, Crabtree RL, Smith DW, Murphy KM, Getz WM (2004). Trophic facilitation by introduced top predators: grey wolf subsidies to scavengers in Yellowstone National Park. J. Anim. Ecol..

