# Supplementary material for: Monitoring canid scent marking in space and time using a biologging and machine learning approach
Source: Sci Rep. 2020 Jan 17;10:588. doi: 10.1038/s41598-019-57198-w (PMC6969016; doi:10.1038/s41598-019-57198-w)
Supplement: Supplementary file 1 — Supplementary Information. [file 41598_2019_57198_MOESM1_ESM.docx]

# Monitoring canid scent marking in space and time using a biologging and machine learning approach: Supplementary Information

Owen R. Bidder*^1^, Agustina di Virgilio^2^, Jennifer S. Hunter^1^, Alex McInturff^1^, Kaitlyn M. Gaynor^1^, Alison M. Smith^3^, Janelle Dorcy^1^, Frank Rosell*^4^

Attachment of accelerometers

The method described in the paper requires that the accelerometer device be attached near enough to the pelvis to detect the tilt produced when animals perform raised-leg urinations or squat postures. For the validation trial that made use of domestic dogs, we designed a simple silastic harness to hold the device in place. This harness, pictured below, was tolerated by all but two of the dogs tested. For some of the larger individuals tested, a nylon belt was also needed to stop the device from slipping backwards.


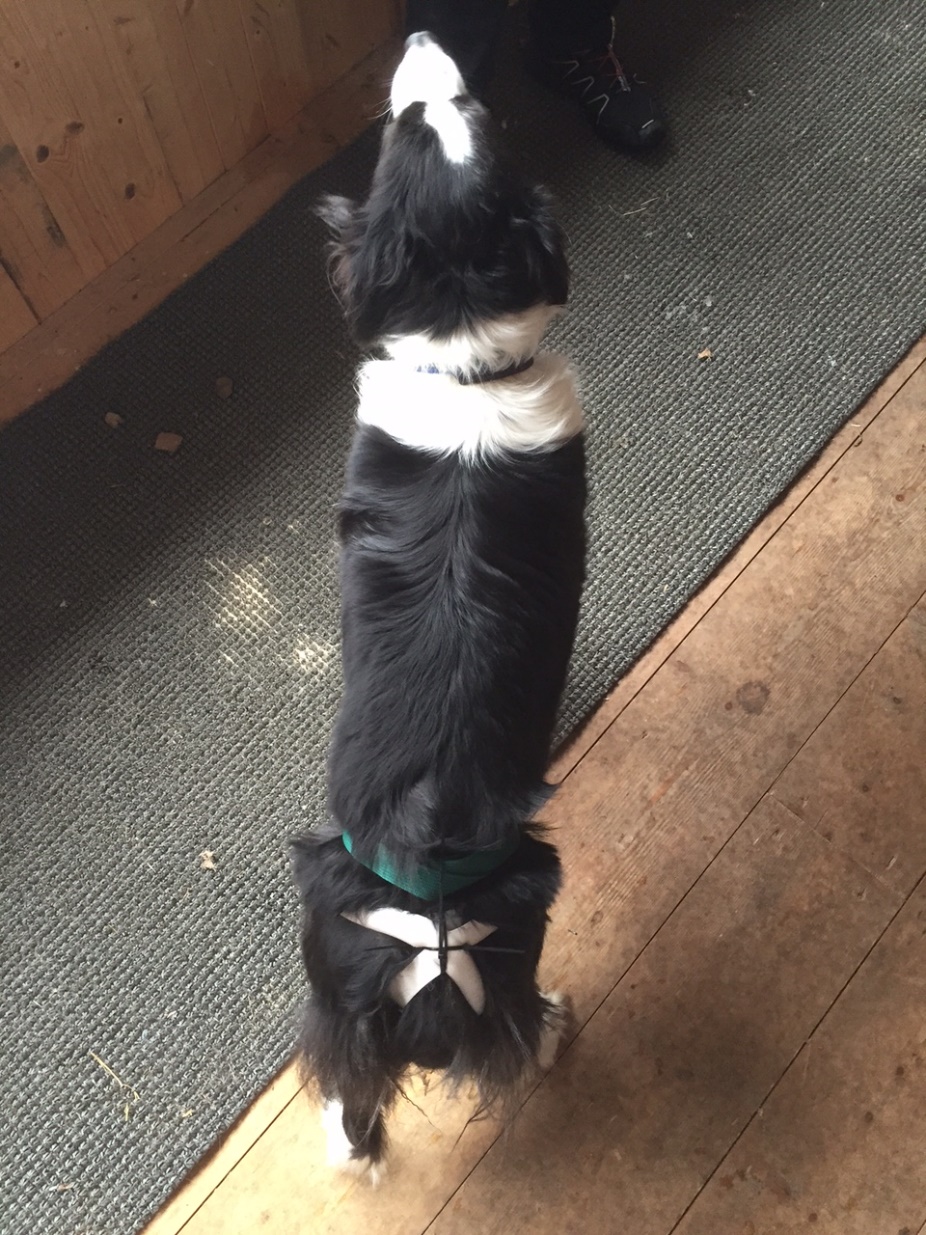


Figure S1: Picture of one of the dogs wearing the silastic harness, pictured from above.

We chose not to use the silastic harness on the guardian dogs because we did not think they would tolerate it for multiple days without supervision, and because we anticipated that free-living animals would not tolerate it (or would bite though it), and so it was important to test an alternative attachment method if the accelerometer method we propose in this study was to be viable. We used a temporary epoxy adhesive to glue the accelerometer to a trimmed area of the fur above the tail, pictured below.


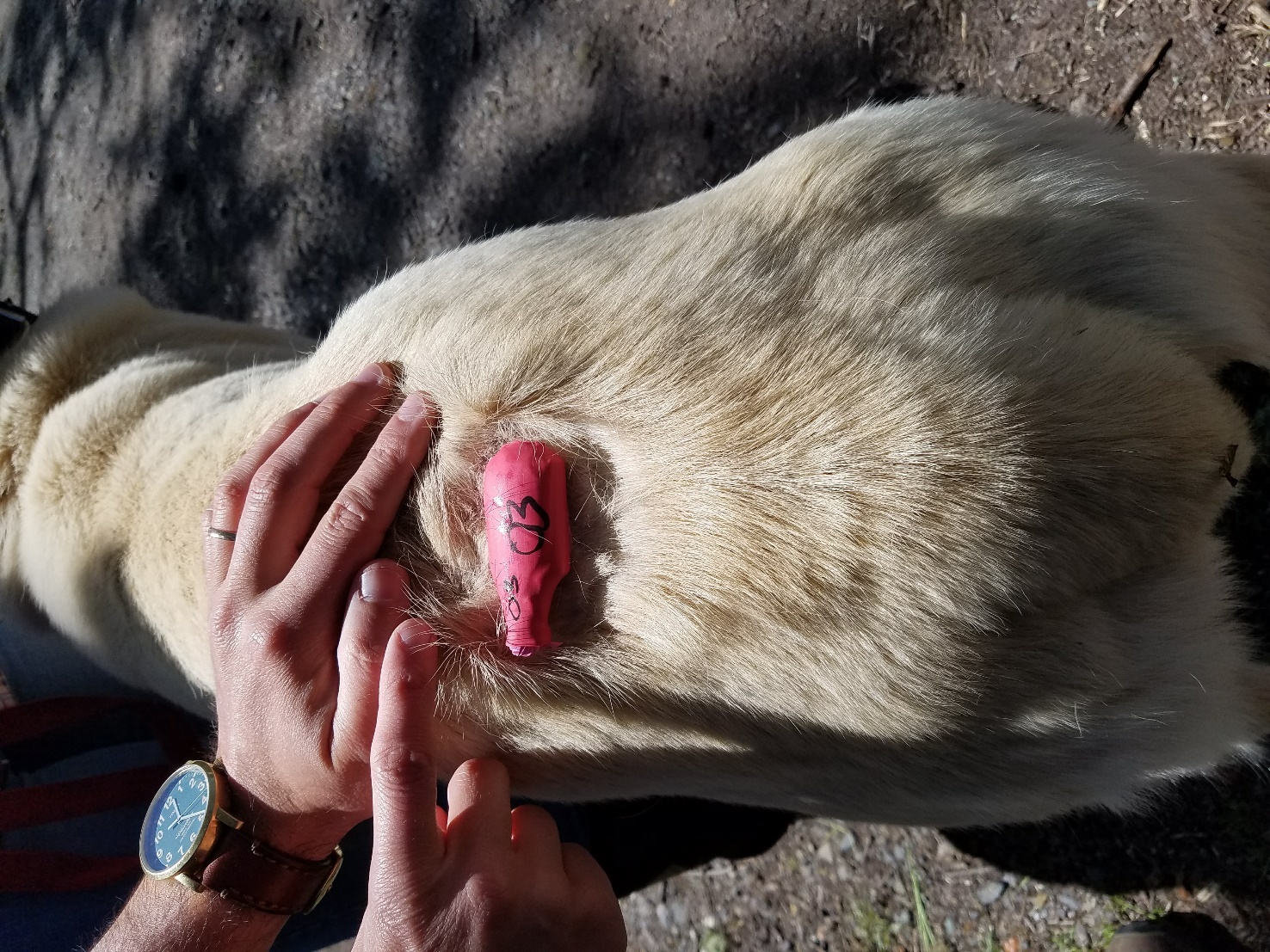


Figure S2: Photo showing device attachment to guardian dog using epoxy adhesive.

The tag was removed by clipping the remaining fur once the deployment was complete. We monitored the site of attachment for injury, but aside from some initial minor reddening of the skin (pictured below), no ill effects were observed.

Although it was easy for us to recapture the domestic dogs used in our study, two of the devices fell off of the guardian dogs prior to retrieval and were never found, resulting in the loss of both data and equipment. As a result of our experience, we recommend that researchers attach a VHF transponder to locate the device once it has detached.


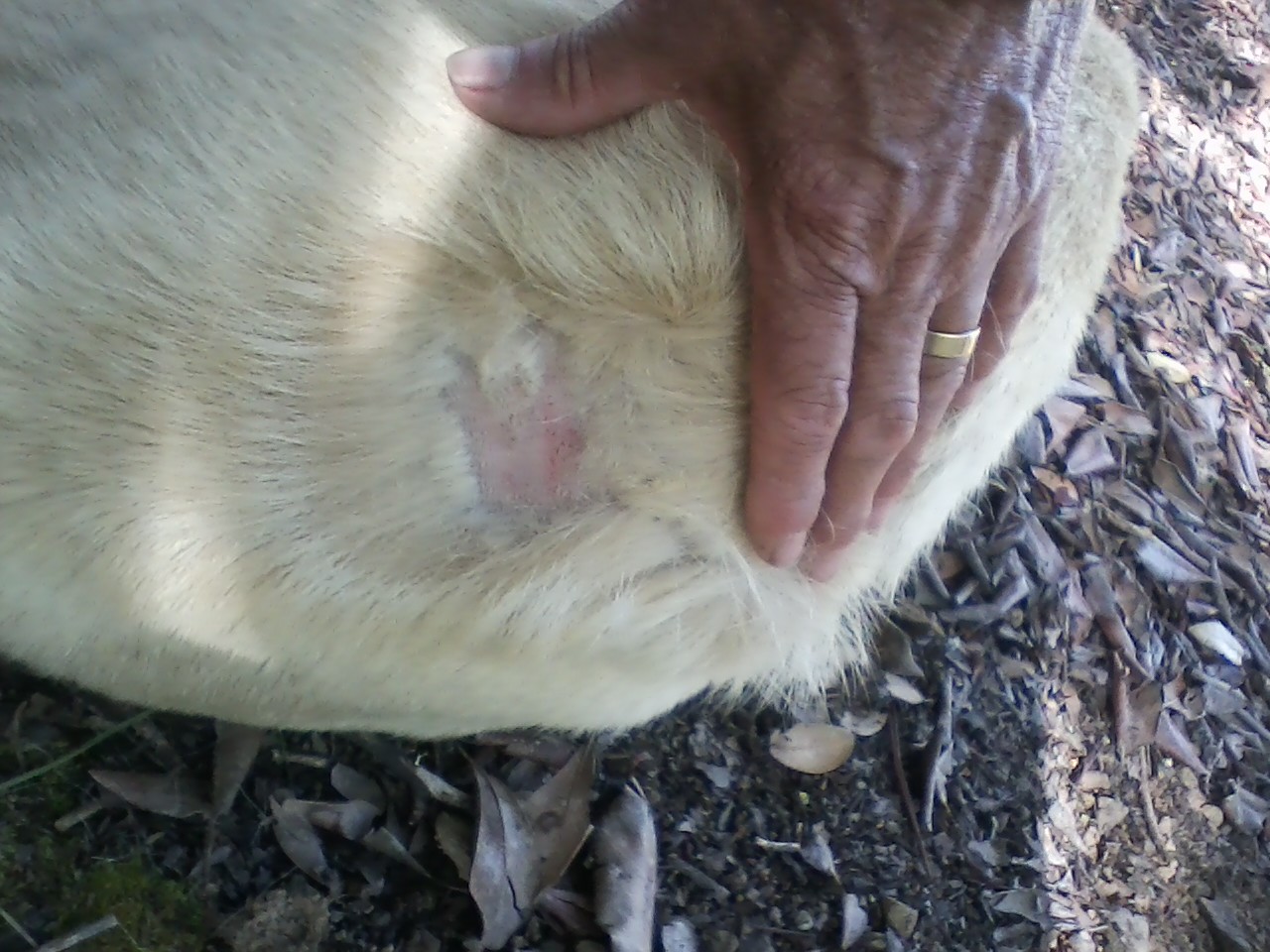


Figure S3: The site of device attachment once the accelerometer was removed. Only minor reddening of the skin was observed.

XYZ Scatter plot of accelerometer data

Within the main article, Figure 1 serves to illustrate how the incidence of scent-marking produces conspicuous changes in the accelerometer data. To provide further illustration, we provide an animated XYZ Scatter plot below. This animation shows how the different behavioural postures populate different regions within the 3D feature space. When new data points are obtained during deployment, they are classified according to their proximity to the clusters that are formed by the training data.

Figure S4: 3D animation of training data for a male dog. 'Other' behavioural data is thinned to highlight the scent-mark related data. Double click in Word to begin the animation.

| Dog  number | Modal  k | Max  Accuracy | Mean  Accuracy | Min  Accuracy | Max  Precision | Mean  Precision | Min  Precision | Max  Recall | Mean  Recall | Min  Recall | Max  F1 | Mean  F1 | Min  F1 | Max  AUC | Mean  AUC | Min  AUC |
| --- | --- | --- | --- | --- | --- | --- | --- | --- | --- | --- | --- | --- | --- | --- | --- | --- |
| 1 | 5 | 0.95 | 0.86 | 0.71 | 0.95 | 0.88 | 0.80 | 0.95 | 0.86 | 0.71 | 0.95 | 0.87 | 0.73 | 0.96 | 0.89 | 0.80 |
| 2 | 9 | 0.92 | 0.84 | 0.62 | 0.92 | 0.88 | 0.85 | 0.92 | 0.84 | 0.62 | 0.92 | 0.85 | 0.68 | 0.95 | 0.88 | 0.76 |
| 3 | 3 | 0.94 | 0.84 | 0.67 | 0.94 | 0.87 | 0.73 | 0.94 | 0.84 | 0.67 | 0.94 | 0.85 | 0.70 | 0.95 | 0.87 | 0.72 |
| 4 | 3 | 0.96 | 0.84 | 0.67 | 0.96 | 0.88 | 0.83 | 0.96 | 0.84 | 0.67 | 0.96 | 0.85 | 0.70 | 0.96 | 0.88 | 0.79 |
| 5 | 5 | 0.90 | 0.73 | 0.48 | 0.92 | 0.83 | 0.66 | 0.90 | 0.73 | 0.48 | 0.90 | 0.76 | 0.52 | 0.91 | 0.77 | 0.47 |
| 6 | 7 | 0.91 | 0.67 | 0.43 | 0.97 | 0.91 | 0.83 | 0.91 | 0.67 | 0.43 | 0.91 | 0.74 | 0.60 | 0.94 | 0.75 | 0.26 |
| 7 | 3 | 0.93 | 0.75 | 0.52 | 0.94 | 0.88 | 0.81 | 0.93 | 0.75 | 0.52 | 0.93 | 0.78 | 0.61 | 0.95 | 0.80 | 0.54 |
| 8 | 5 | 0.96 | 0.74 | 0.52 | 0.96 | 0.89 | 0.84 | 0.96 | 0.74 | 0.52 | 0.96 | 0.79 | 0.61 | 0.96 | 0.82 | 0.69 |
| 9 | 5 | 0.93 | 0.82 | 0.57 | 0.93 | 0.88 | 0.81 | 0.93 | 0.82 | 0.57 | 0.93 | 0.84 | 0.63 | 0.95 | 0.87 | 0.73 |
| 10 | 3 | 0.95 | 0.79 | 0.59 | 0.95 | 0.89 | 0.84 | 0.95 | 0.79 | 0.59 | 0.95 | 0.82 | 0.69 | 0.96 | 0.85 | 0.74 |
| 11 | 3 | 0.95 | 0.79 | 0.59 | 0.95 | 0.89 | 0.84 | 0.95 | 0.79 | 0.59 | 0.95 | 0.82 | 0.69 | 0.96 | 0.85 | 0.74 |
| 12 | 3 | 0.93 | 0.83 | 0.73 | 0.94 | 0.87 | 0.83 | 0.93 | 0.84 | 0.73 | 0.93 | 0.84 | 0.74 | 0.94 | 0.88 | 0.79 |
| 13 | 3 | 0.89 | 0.73 | 0.46 | 0.90 | 0.83 | 0.69 | 0.89 | 0.73 | 0.46 | 0.90 | 0.75 | 0.54 | 0.92 | 0.77 | 0.38 |
| 14 | 3 | 0.90 | 0.81 | 0.71 | 0.90 | 0.87 | 0.83 | 0.90 | 0.81 | 0.71 | 0.90 | 0.83 | 0.74 | 0.90 | 0.86 | 0.80 |
| 15 | 3 | 0.91 | 0.80 | 0.66 | 0.92 | 0.86 | 0.76 | 0.91 | 0.80 | 0.67 | 0.91 | 0.81 | 0.69 | 0.92 | 0.85 | 0.73 |

Table S1: Details of the results of the pairwise surrogacy analysis. Models were trained on each male dog and applied to predict scent marking of all others in a pair wise fashion. Here we present the maximum, mean and minimum of the 14 pairwise comparisons for each surrogate.

Directions for use of Python scripts

To encourage uptake of the methods described in this study, we have provided all of the code necessary to implement detection of scent-marking detection in accelerometer data, and link those detected events to GPS data collected concurrently. This code is presented in a Jupyter Notebook on a GitHub Repository, which can be found at <http://bit.ly/scent-marking> . We hope that this file format provides a convenient means to follow the analysis steps.

Some devices may produce data files that follow a different format, but the code should be general enough to implement for most devices with some minor adjustment to the code. We encourage any researchers interested in implementing the code on their own data to reach out to the corresponding authors.

A brief overview of the workflow is included below;

1. When using the AX3 accelerometer, use OMGui (<https://github.com/digitalinteraction/openmovement/wiki/AX3-GUI>) to convert the .cwa files in to a resampled CSV file. We down-sampled data to 25 Hz to improve computation speed.
2. Do a quick summary plot using the crop_file.py. We used this script to crop the file, as the device continued to record after it was removed from the dogs, producing large files with superfluous data. Use the ‘chunksize’ argument to specify how many rows should be taken from the start of the file.
3. Before we can use the KNN to predict scent-marking events, we need to use an initial period of observation to train the model. In our study, we obtained a training set by observing the dog prior to release. Thus, the accelerometer file must be split in to training and testing periods. The next few steps detail how one may prepare and train the KNN.
4. We must synchronize the timestamp of the accelerometer data with the video that details to behavioural observations of the dog. At the beginning of the observation period, we performed conspicuous calibration tilts (the device was rotated 90 degrees for 5 second, and this was repeated 3 times). With this in mind, use subsample.py to plot 10-minute summaries of the data. The function will run through the first 3 days of the file (after that the tag was off the animal and so it's just excess data), showing a 10 minute window in each plot to help you find the calibration tilts mentioned previously. This function needs there to be a folder called 'explore', which will be filled with plots for you to run through sequentially (using Windows’ Photo app for instance). In future, we will implement a more elegant interactive plot for Users to use in Python, but this is currently in development. Once the tilts are identified and their time in the accelerometer file noted, you can trim the start of the file to ca. 5 seconds before the tilts, to make them easy to spot and sync to the video in ELAN.
5. Load the trimmed data in to ELAN, along with the video. Sync the two using the procedure detailed in the video tutorial kindly produced by Cassim Ladha (<https://www.youtube.com/watch?v=zofLvUU0Gus>). Then run through the video and make annotations. Following this, output annotations as a tab delimited text file to load back in to Python.
6. Using the label_accl.py script, load the accelerometer data and the annotation record, combine the two to produce one annotated accelerometer dataset. This will output an accelerometer file with an extra column that contains the class of behaviour being undertaken by the animal at that time.
7. Use the KNN_sm.py script to actually train and test the KNN classifier to the annotated data. Functions to obtain performance metrics (e.g. Accuracy) are included, along with a means to construct confusion matrices to evaluate the KNN’s performance.
8. Use the smooth_knn.py script to smooth the KNN predictions to 1 Hz so that they can be linked to GPS locations recorded at that time. This script takes the 25 Hz accelerometer data and smooths it to 1 Hz, by taking the modal class for each 25-row group that constitutes a second of accelerometer data. There is also a function in this script to check each class' accuracy and ensure the smoothed data matches the actual classes as recorded by video (after those annotations too has been smoothed).
9. Using the link_gps.py script, merge the predicted classes of the accelerometer data (a product of the KNN) to the Latitude and Longitude values in the GPS data. Once the GPS data has behavioural classes, identify the points where dogs are making left and right scent marks. These points then can be written to file for use in GIS software or R for further analysis.
10. These steps can then be repeated using data from the unobserved period (obtained during deployment on free living animals for instance) to obtain a record of scent-marking locations. The necessary code segments are presented in the scripts above too.
